# Three mitochondrial genomes of *Pseudogobio* fishes (Cypriniformes: Gobionidae)

**DOI:** 10.1080/23802359.2020.1797553

**Published:** 2020-07-29

**Authors:** Junwei Fu, Cuizhang Fu

**Affiliations:** Ministry of Education Key Laboratory for Biodiversity Science and Ecological Engineering, Coastal Ecosystems Research Station of the Yangtze River Estuary, Institute of Biodiversity Science and Institute of Eco-Chongming, School of Life Sciences, Fudan University, Shanghai, China

**Keywords:** *Pseudogobio*, Cypriniformes, Gobionidae, *Pseudogobio guilinensis*, East Asia

## Abstract

New mitochondrial genomes of *Pseudogobio guilinensis*, *P. giganteus*, and *P. anderssoni* have the length of 16,605, 16,606, and 16,609 bp with A + T bias. Inferred phylogeny shows that *P. guilinensis* occupies basal position. *P. esocinus* and *P. anderssoni*+*P. longirostris* are sister groups, and they together are a sister taxa of *P. giganteus*.

*Pseudogobio* fishes (Cypriniformes: Gobionidae) are a group of freshwater small-sized fishes (<15 cm), and they are distributed in East Asia (Tominaga and Kawase 2019; Fu 2020). The genus *Pseudogobio* has been revised in a recent study (Fu 2020), and they are comprised of eight valid nominated species and two cryptic species. Mitochondrial genomes of three species *P. guilinensis*, *P. giganteus*, and *P. anderssoni* have been determined in this study, and they would be used to delimit *Pseudogobio* species and to infer their phylogeny.

*Pseudogobio guilinensis* (voucher FDZM-PGuPingL20170716-01) is collected from the Pingle County, China (24.64°N, 110.65°E), *P. anderssoni* (FDZM-PAAnY20180728-01) from the Anyi County, China (28.84°N, 115.56°E), and *P. giganteus* (FDZM-PGiXYing20170921-01) from the Haikou City, China (24.64°N, 110.65°E). These specimens have been deposited in the Zoological Museum of Fudan University (FDZM). The Sanger sequencing is used to obtain mitochondrial genomes.

Three new mitochondrial genomes consist of 13 protein-coding genes, two rRNA genes, 22 tRNA genes, and one control region. Their lengths are 16,605, 16,606, and 16,609 bp with A + T bias of base compositions 56.8, 57.8, and 57.5%. Among 13 protein-coding genes, GTG is the start codon of COI gene, and ATG is start codons of the remaining 12 genes. Four kinds of stop codons are revealed to include TAG (ATP8, ND5), TAA (ND1, COX1, ATP6, ND4L, ND6), T— (COX2, COX3, CYTB), and TA– (ND2, ND3, ND4). Six pairs of adjoining genes show 1–7 bp of gene overlaps and 13 pairs of adjoining genes show 1–31 bp of gene intervals. Patterns in gene arrangements, codon uses, gene overlaps, and gene intervals are consistent with published fish mitochondrial genomes (Li et al. 2018; Tong and Fu 2019; Zhang and Fu 2019).

Phylogenetic relationships between *Pseudogobio* fishes and their close relatives are inferred using five partitions (each codon of all protein-coding genes, 12S + 16S rRNA genes, and all tRNA genes) of 28 mitochondrial genomes in the software IQ-TREE 1.6.2 (Nguyen et al. 2015). Our tree (Figure 1) reveals that the genus *Pseudogobio* is a monophyletic group. *P. guilinensis* is located in basal position. *P. esocinus* and *P. anderssoni* + *P. longirostris* are sister groups, and they together are a sister taxa of *P. giganteus*.

**Figure 1.**
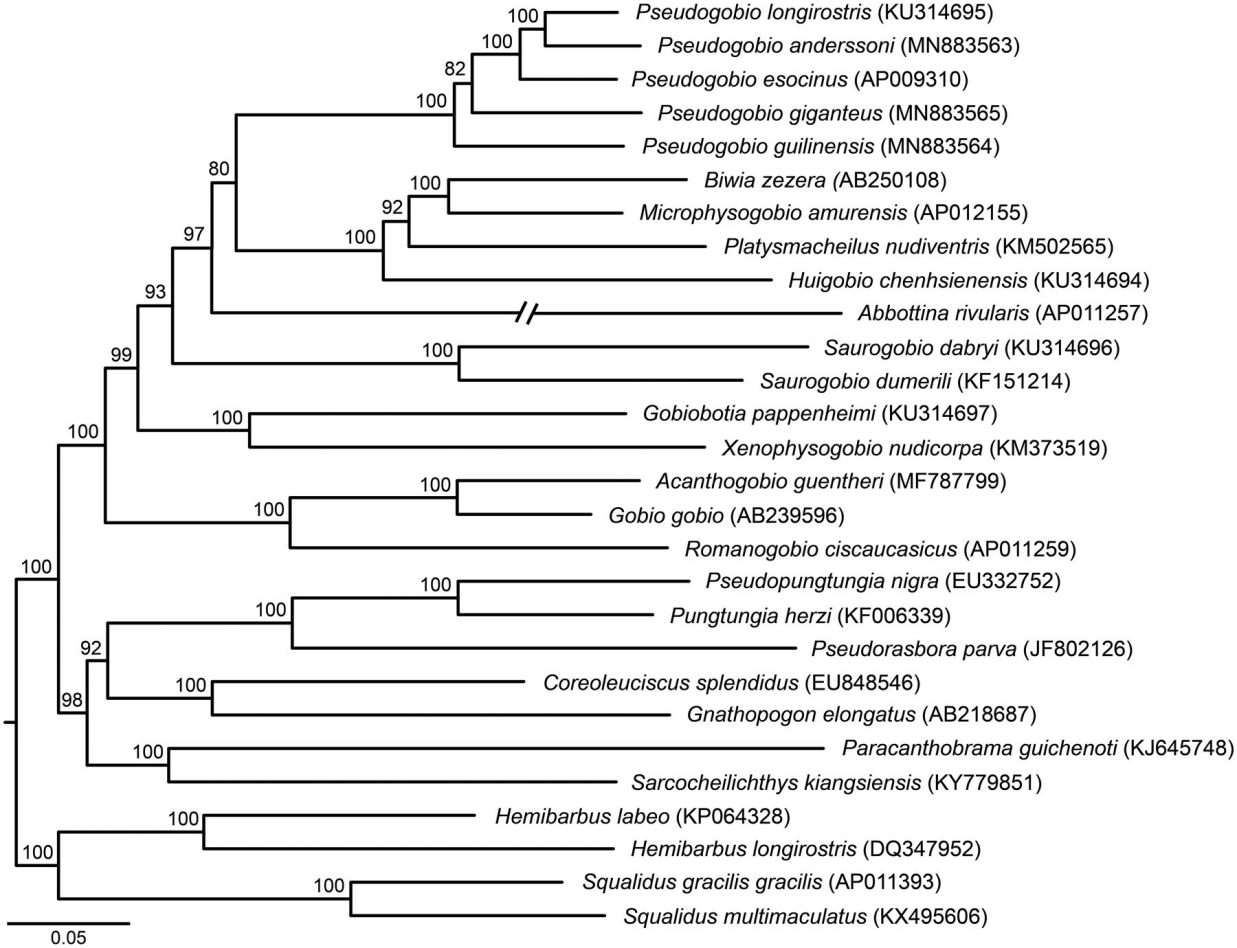
A maximum likelihood tree of *Pseudogobio* fishes and their close relatives using 28 mitochondrial genomes under the software IQ-TREE. Bootstrap confidences are shown above branches. GenBank numbers are listed in the parentheses.

## Data Availability

Three new mitochondrial genomes have been released in the GenBank with accession numbers as follows: MN883563 (https://www.ncbi.nlm.nih.gov/nuccore/MN883563); MN883564 (https://www.ncbi.nlm.nih.gov/nuccore/MN883564); MN883565 (https://www.ncbi.nlm.nih.gov/nuccore/MN883565).
